# Tubulointerstitial Nephritis and Uveitis in a Pediatric Patient

**DOI:** 10.7759/cureus.66380

**Published:** 2024-08-07

**Authors:** Madalena Carvalho, Francisca Galhardo Saraiva, Inês Coutinho, Vanda Bento, Marta Cabral

**Affiliations:** 1 Pediatric Service, Child and Youth Department, Hospital Professor Doutor Fernando Fonseca, Lisboa, PRT; 2 Ophthalmology Service, Hospital Professor Doutor Fernando Fonseca, Lisboa, PRT; 3 Pediatric Rheumatology, Child and Youth Department, Hospital Professor Doutor Fernando Fonseca, Lisboa, PRT

**Keywords:** renal biopsy, inflammation, autoimmune disease, uveitis, interstitial nephritis

## Abstract

Tubulointerstitial nephritis and uveitis syndrome (TINU) is a rare autoimmune disease. It is characterized by uveitis and kidney damage. The presentation of uveitis is typically anterior and bilateral, while the renal lesion is an acute interstitial nephritis. We report a case of an adolescent diagnosed with this disease, who presented with ocular and constitutional symptoms. An ophthalmologic examination confirmed the diagnosis of uveitis, and subsequent systemic evaluation revealed impaired renal function. The findings of the renal biopsy established the diagnosis after ruling out other systemic diseases. Given the rarity and nonspecific clinical presentation of this condition, a high level of suspicion is required for early diagnosis and treatment. Clinicians should consider this diagnosis in a pediatric patient with uveitis and impaired renal function.

## Introduction

Tubulointerstitial nephritis and uveitis (TINU) syndrome is a rare, inflammatory disease, first described in 1975 [1,2]. To date, more than 250 cases have been reported [3]. It is characterized by uveitis and kidney injury, with a higher prevalence in young people and female predominance [2,4,5]. It is an immune-mediated disease that can be triggered by drugs or infections, but, in most cases, there is no defined etiology (idiopathic) [2]. The cell-mediated immune response is thought to be the primary driver of inflammation [6]. The diagnosis is suggested by the association of uveitis with kidney injury, with a renal biopsy consistent with acute interstitial nephritis [3]. To establish the diagnosis, it is also necessary to exclude infections and systemic inflammatory diseases that could explain both manifestations [1,2]. The renal biopsy typically shows interstitial edema with inflammatory cell infiltrates (T lymphocytes, neutrophils, plasma cells) and tubular injury with tubular edema. Eosinophils and noncaseating granulomas may be present, and the glomeruli and vascular structures are usually preserved [3]. Uveitis is anterior and bilateral in most patients [5,7]. Regarding disease progression, renal damage usually resolves spontaneously with recovery of renal function; however, uveitis may persist or recur after the initial presentation of the disease [1]. Diagnosing this condition can be challenging as renal and ocular involvement may not occur at the same time [1].

## Case presentation

A 16-year-old adolescent male with a history of epilepsy, treated with sodium valproate, presented to the emergency department with blurred vision, ocular pain, photophobia, and unilateral conjunctival hyperemia of one month's duration. Furthermore, he also reported asthenia and a 10% weight loss over three months. The patient denied the presence of any of the following symptoms: fever, arthralgia, myalgia, rash, respiratory symptoms, gastrointestinal symptoms, xerophthalmia, xerostomia, and oral or genital ulcers. He had no history of contact with animals or individuals with specific infectious diseases and he had not travelled to rural areas or tropical countries.

The ophthalmological evaluation revealed a best-corrected visual acuity of 20/20 in the right eye and 20/25 in the left eye, with mild, non-granulomatous anterior uveitis in the left eye. The patient was treated with topical corticosteroids and tropicamide. One week later, there was no response, and the condition progressed to bilateral anterior uveitis. As this was an adolescent with uveitis, a systemic investigation was performed.

The laboratory evaluation (Table 1) revealed normocytic normochromic anemia, increased inflammation parameters, mild thrombocytosis, and kidney function impairment (creatinine 1.45 mg/dL, urea 26 mg/dL, glomerular filtration rate 86.8 mL/min). The urinalysis showed non-nephrotic proteinuria (13.6 mg/m^2^/hour), and the urine sediment had some leukocytes, rare erythrocytes, and a leukocyte cast. The infectious study showed no significant changes (Table 2), and the autoimmune investigation (Table 3) was positive for antinuclear antibodies (titer 1:160), with other antibodies negative. Protein electrophoresis showed hypergammaglobulinemia, and anti-streptolysin O antibody (ASLO) was elevated.

**Table 1:** Laboratory results AST, aspartate aminotransferase, ALT, alanine aminotransferase; FT4, free thyroxine; TSH, thyroid-stimulating hormone

Laboratory results	Values	Reference range
Hemoglobin	8.3 g/dL	13–17 g/dL
Mean corpuscular volume	84.7 fL	78-96 fL
White blood cells	10,3 x 10^9^/L	4–10 x 10^9^/L
Platelets	461 x 10^9^/L	150–400 x 10^9^/L
C-reactive protein	3.63 mg/dL	<0.50 mg/dL
Erythrocyte sedimentation rate	125 mm/h	<13 mm/h
Ferritin	337 ng/mL	14–152 ng/mL
AST	15 U/L	<40 U/L
ALT	9 U/L	<41 U/L
Creatinine	1.45 mg/dL	0.7–1.20 mg/dL
Urea	26 mg/dL	18–45 mg/dL
Proteinuria	13.6 mg/m^2^/hour	-
urinary β2 microglobulin	49.73 mg/L	<0.30 mg/L
FT4	0.90 ng/dL	0.98–1.63 ng/dL
TSH	1.300 mUI/L	0.51–4.3 mUI/L

**Table 2:** Infectious study Ab, antibody; ASLO, anti-streptolysin O antibody; CMV, cytomegalovirus; HCV, hepatitis C virus; HIV, human immunodeficiency virus; VDRL, Venereal Disease Research Laboratory

Infectious study	Results	Reference range
Ab anti-HIV 1 and 2/Ag p24	Non-reactive	-
AgHbs	Non-reactive	-
Anti-HCV	Non-reactive	-
Ab *Borrelia burgdorferi *IgM/IgG	Non-reactive	-
Ab anti-*Bartonella hensellae *IgM	Non-reactive	-
Ab anti-*Bartonella hensellae* IgG	Non-reactive	-
Ab Toxocara spp larva migrans	2	<11.00
Ab anti-*Toxoplasma gondii *IgM	Negative	-
Ab anti-*Toxoplasma gondii *IgG	Negative	-
VDRL	Negative	-
Ab anti-virus Epstein Barr VCA IgG	Positive (>750 U/mL)	-
Ab anti-virus Epstein Barr VCA IgM	Negative	-
Ab anti-CMV IgG	Positive (84.50 UI/mL)	-
Ab anti-CMV IgM	Negative	-
ASLO	481 UI/mL	<214 UI/mL

**Table 3:** Autoimmune study Ab, antibody; ACE, angiotensin-converting enzyme; ANA, antinuclear antibodies; ANCA, anti-neutrophil cytoplasm antibodies; C3, complement 3; C4; complement 4; HLA, human leukocyte antigen; RF, rheumatoid factor

Autoimmune study	Results	Reference range
ANA	Positive (titer 1:160)	-
HLA-B27	Negative	-
RF	<10 UI/mL	-
Ab anti-*Saccharomyces cerevisiae* IgA	Negative	-
Ab anti-*Saccharomyces cerevisiae* IgG	Negative	-
ANCA	Negative	-
Anti-dsDNA	Negative	-
Ab anti-SSA	Negative	-
Ab anti-SSB	Negative	-
ACE	31.04 U/L	8–52 U/L
C3	189 mg/dL	90–180 mg/dL
C4	34.6 mg/dL	10–40 mg/dL
HLA-B27	Negative	-
Immunoglobulin G	3,002 mg/dL	549–1,584 mg/dL
Immunoglobulin A	439.86 mg/dL	61–348 mg/dL
Immunoglobulin M	81.57 mg/dL	23–259 mg/dL

The patient was evaluated by a cardiologist, who ruled out cardiac involvement. Renal ultrasound identified hyperechoic areas in the renal parenchyma of both kidneys, suggestive of medical nephropathy. A thoraco-abdomino-pelvic computed tomography scan showed no abnormalities and ruled out lymphoproliferative disease. Further laboratory investigation revealed an elevated urinary B2 microglobulin level (49.73 mg/dL). Therefore, a presumptive diagnosis of TINU syndrome was made.

**Figure 1:**
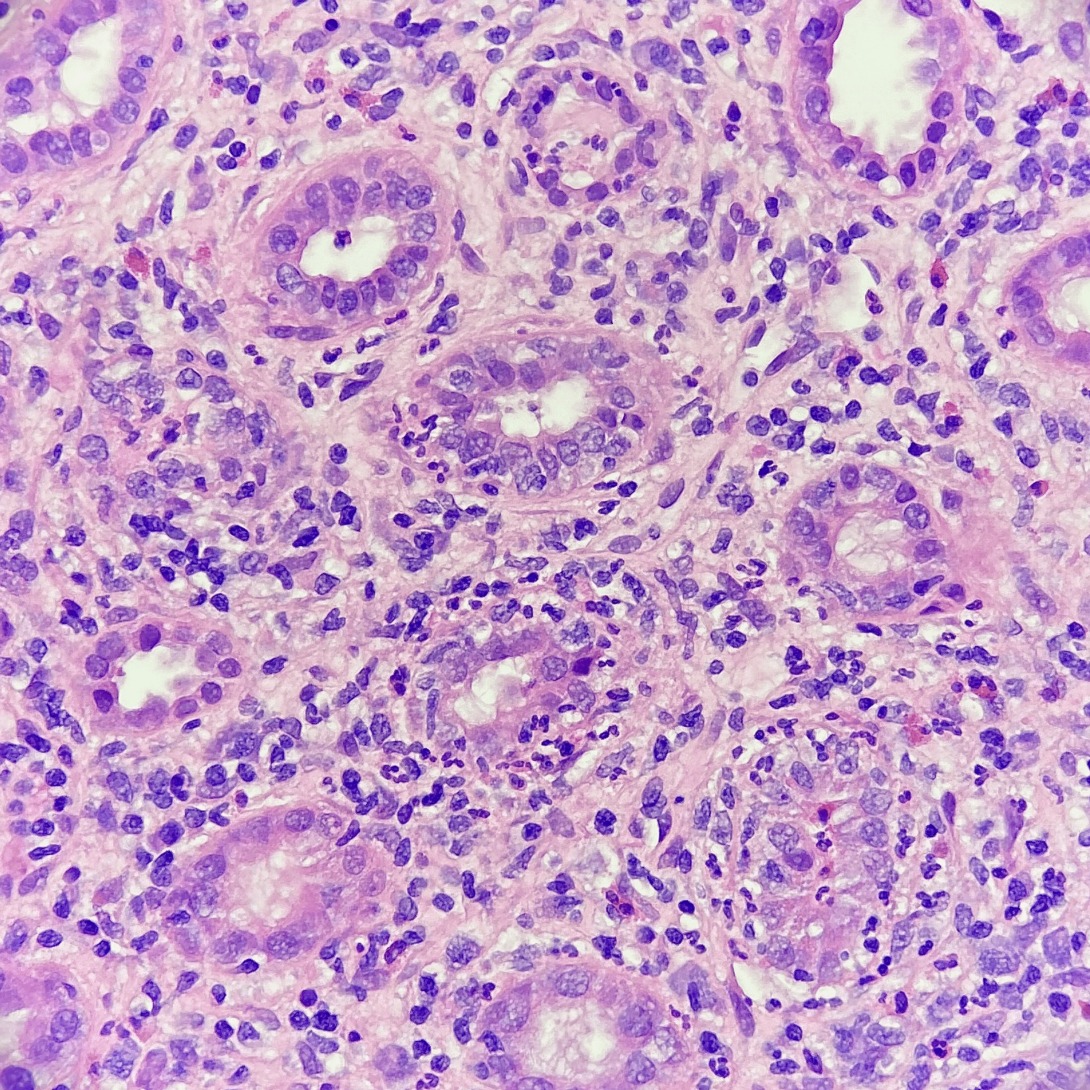
Renal biopsy The biopsy showed proximal and distal tubules with degenerative and reactive changes, permeated by lymphocytes and neutrophils, with associated basement membrane rupture; intense, predominantly chronic inflammatory infiltrate, rich in lymphocytes and plasma cells, with associated neutrophils and eosinophils; and marked edema in the peri-tubular stroma.

A renal biopsy was then performed (Figure 1), which showed preserved glomeruli; proximal and distal tubules with degenerative and reactive changes, permeated by lymphocytes and neutrophils, with associated basement membrane rupture; an intense, predominantly chronic inflammatory infiltrate, rich in lymphocytes and plasma cells, with associated neutrophils and eosinophils; and marked edema in the peri-tubular stroma. The biopsy results corroborated the diagnosis of tubulointerstitial nephritis (TINU), and the patient was discharged after 12 days with oral prednisolone (1 mg/kg/day) and topical dexamethasone.

The patient subsequently attended follow-up appointments with the nephrology, ophthalmology, and rheumatology. Regarding renal involvement, the clinical course was favorable, with renal function normalizing within four weeks and no recurrences. However, the uveitis had four relapses over a two-year period. The first occurred due to poor adherence and non-compliance with the prescribed treatment, and the second recurrence occurred following corticosteroid tapering and the patient started methotrexate at that moment. The remaining two relapses were due to non-adherence to treatment and during systemic therapy tapering again.

## Discussion

This report presents the case of a male adolescent diagnosed with TINU. TINU is a rare disease, probably underdiagnosed [1], with a higher prevalence in young women, but it can affect a wide spectrum of patients [4].

Patients may be asymptomatic or present constitutional symptoms [4], as observed in the presented case. Anemia and increased inflammatory parameters (such as ferritin and erythrocyte sedimentation rate) are laboratory findings that may be present in this immune-mediated disease [3].

A diagnosis of TINU requires the exclusion of other infectious and systemic diseases that could explain the renal and ocular manifestations [1,2] as there are no specific laboratory markers for this condition [3]. In the presented case, infectious causes were ruled out. In the autoimmune study, there were positive antinuclear antibodies (ANA) (titer 1:160), with the remaining autoimmune markers negative and a clinical picture not suggestive of an autoimmune-specific disease. The disease has been associated with the presence of anti-neutrophil cytoplasm antibodies (ANCA), ANA, rheumatoid factor (RF), and hypocomplementemia [3]. Cardiac evaluation was normal, and the thoraco-abdomino-pelvic CT scan ruled out lymphoproliferative disease. These examinations allowed the exclusion of other systemic diseases that could explain the renal and ocular inflammation.

The majority of patients (80%) with this syndrome present with non-granulomatous anterior uveitis of sudden onset, characterized by symptoms of red eye, ocular pain, and photophobia [4]. Additionally, bilateral ocular involvement is observed in the majority of cases. Initially, uveitis is commonly unilateral, with subsequent progression to bilateral involvement (within a median time of one week) [4], as was observed in our patient. Uveitis can precede, occur simultaneously, or follow renal disease [7].

The kidney injury is acute interstitial nephritis. Patients may present with flank pain, sterile pyuria, hematuria, proteinuria (usually subnephrotic), and impaired kidney function [3]. The urinary sediment may contain red cells and red cell casts [2]. Tubular proteinuria may be detected, but elevated levels of albuminuria are rarely present, as the condition does not involve glomerular injury [2]. In TINU, renal involvement is generally mild and may resolve spontaneously [4]. A prospective cohort study including 45 patients (aged ≤22 years) with uveitis concluded that urinary β2 microglobulin and serum creatinine are sensitive markers for the detection of renal dysfunction to diagnose TINU syndrome in young patients [8]. β2 microglobulin is a small protein that is filtered by the glomeruli into the ultrafiltrate and then reabsorbed by the renal tubules. In cases of tubulointerstitial nephritis, β2 microglobulin is filtered but not reabsorbed, leading to elevated levels of this protein in the urine [8]. Urinary β2 microglobulin levels can remain elevated for a long period after kidney injury, even after creatinine normalization. However, it is important to recognize that these markers lack specificity for TINU and can be altered in tubulointerstitial nephritis of any etiology [6]. In the presented case, the patient exhibited typical laboratory abnormalities indicative of kidney involvement in this condition, such as increased creatinine, non-nephrotic proteinuria, and elevated urinary β2 microglobulin. The renal biopsy findings were also consistent with the diagnosis.

In summary, the key features for the diagnosis of TINU are the presence of an anterior uveitis and evidence of tubulointerstitial nephritis. Although an anterior/intermediate or panuveitis may be present, anterior chamber inflammation should be present. Tubulointerstitial nephritis is best diagnosed by renal biopsy, but it can also be inferred from other renal and urinary findings such as elevated serum creatinine, abnormal urinalysis, and elevated urine β2 microglobulin [9].

In terms of treatment, topical and systemic corticosteroids are indicated as first-line therapy for uveitis. However, it is important to note that relapses and recurrences are common in ocular inflammation [3,10]. In some cases, drugs such as methotrexate, cyclosporine, and/or mycophenolate mofetil may be necessary as corticosteroid-sparing immunosuppressants [3]. The renal injury associated with TINU is typically self-limiting [3,10], but patients with progressive kidney function impairment are usually treated with prednisolone [3]. While renal function often normalizes in pediatric patients and is not typically a primary driver of treatment management, long-term kidney injury can occur even years after the initial insult, requiring long-term monitoring [6]. In this case, the adolescent initially demonstrated a favorable response to both topical and systemic corticosteroid therapy. However, as described in the literature, it became necessary to initiate therapy with methotrexate due to poor control of ocular inflammation.

## Conclusions

We presented this case to emphasize the importance of considering TINU as a potential diagnosis in patients with uveitis and renal injury, particularly in the presence of anterior uveitis and evidence of tubulointerstitial nephritis. In a young patient with uveitis (typically anterior, bilateral, non-granulomatous), even in the first episode, a systemic evaluation with laboratory tests, including renal function and urinalysis, is essential. In such cases, the possibility of TINU should be considered.

Due to its rarity, this condition presents a diagnostic challenge. A high level of suspicion is necessary for early diagnosis and subsequent treatment, which are essential for a favorable prognosis. Furthermore, we highlight the importance of a multidisciplinary approach for proper follow-up.
